# Protein biomarker profiles in serum and CSF in 158 patients with PTLDS or persistent symptoms after presumed tick-bite exposure compared to those in patients with confirmed acute neuroborreliosis

**DOI:** 10.1371/journal.pone.0276407

**Published:** 2022-11-03

**Authors:** Kenneth Nilsson, Elisabet Skoog, Marie Edvinsson, Andreas Mårtensson, Björn Olsen

**Affiliations:** 1 Department of Medical Sciences, Section of Infectious Diseases, Uppsala University, Uppsala, Sweden; 2 Department of Medical Sciences, Section of Clinical Microbiology, Uppsala University, Uppsala, Sweden; 3 Department of Women’s and Children’s Health, International Maternal and Child Health, Uppsala University, Uppsala, Sweden; 4 Department of Medical Sciences, Zoonosis Science Centre, Uppsala University, Uppsala, Sweden; University of Kentucky College of Medicine, UNITED STATES

## Abstract

**Background:**

Current diagnostics for patients with lingering symptoms categorized as post-treatment Lyme disease syndrome (PTLDS) have their limitations and may be difficult to interpret. The aim of this exploratory study was to evaluate the feasibility of protein biomarker profiling as a diagnostic platform for this category of patients and to compare these results with similarly obtained results from a group of patients with acute neuroborreliosis.

**Methods and findings:**

Two groups of patient cohorts (Cohort 1 and 2) were analyzed for biomarkers in serum and cerebrospinal fluid (CSF); the results were used for group-level comparison. Cohort 1 comprised 158 adult patients selected from 224 previously diagnosed patients, who between October 2015 and December 2018, after referral, were enrolled and structurally investigated based on defined inclusion criteria. They displayed similar lingering symptoms, with a duration of at least 6 months, after presumed previous tick-borne infection (TBI) and are fully described in a previously published study originating from the Center for Vector-borne Infections (CVI), Uppsala University Hospital, Sweden. Cohort 2, comprised 30 patients diagnosed at Uppsala University Hospital between 2016 and 2019 with laboratory-confirmed acute neuroborreliosis. Their proteomic results, based on serum and CSF analyses, were compared with the 158 patients in Cohort 1. The expression and the concentration of potential biomarkers in each patient’s serum and CSF samples were measured based on two multiplex protein panels enabling simultaneous analysis of 92 inflammatory and neurology biomarkers. The PTLDS patient subgroup showed no nominally significant proteins compared to the other CVI patients in Cohort 1. However, CVI patients with signs of inflammation, which were evenly distributed in Cohort 1, showed 16 significantly (p <0.05) different proteins in both CSF and serum, but no association was seen with laboratory-confirmed exposure to *Borrelia* spp or other TBIs. When comparing the two cohorts, different protein profiles were observed, with 125/148 significantly different proteins in CSF and 93/174 in serum, in patients with laboratory confirmed acute neuroborreliosis, of which 6 in CSF and 6 in serum were significant at the p <0.001 level.

**Conclusions:**

In this first comprehensive inflammatory and neurological biomarker profile study no differences in biomarker profiles were detected between patients with PTLDS and patients with similar persisting symptoms but who did not meet the PTLDS criteria, regardless of whether laboratory verified previous exposure to *Borrelia* or other TBI’s were present. However, the expressed markers differed from those found in patients with confirmed acute neuroborreliosis, which does not support the view that PTLDS reflects an ongoing *Borrelia* infection. Further studies are needed to understand and assess the usefulness of biosignatures of patients with PTLDS before they can be applied in a clinical setting.

## Introduction

Proteins play key roles in various biological processes, including defense against infection [1]. New multiplexed technology has made it possible to measure many target biomarkers quantitatively; it has the advantage of minimal cross-reactivity compared to benchmark multiplexing platforms [2]. Lyme disease (LD) is an increasingly important public health concern [3, 4]. The disease is caused by infection with *Borrelia burgdorferi* s.l. following a bite from an infected tick [5]. Symptoms of early acute LD can include erythema chronicum migrans (ECM) with or without systemic symptoms such as fever and malaise. Signs of disseminated infection may occur early or late in the disease process and can involve the skin, certain neurologic and cardiac manifestations, and arthritis, all of which usually respond well to conventional antibiotic therapy [5, 6]. Various reports have suggested that 2 to 40% of appropriately treated patients may subsequently suffer from persisting, primarily patient-reported minor to severe symptoms, including fatigue, musculoskeletal or neurocognitive symptoms, lasting for months or even years [7–10]. This lingering illness, called post-treatment Lyme disease syndrome (PTLDS), is distinct from what has been described for other subsets of patient categories who attribute their symptoms to chronic borreliosis, also called chronic Lyme; these subsets exhibit symptoms of unknown cause, with or without objective clinical findings consistent with LD, and have become topics of ongoing controversy and debate [11].

The pathology of LD is the result of complex vector-pathogen-host interactions and the different manifestations of host immune responses to pathogen invasion, dissemination, and persistence [12]. The high or even rising incidence of LD demands a more complete understanding of the disease process, particularly the disease mechanisms underlying long-term outcomes of *B*. *burgdorferi* infection such as PTLDS [13]. Since 2015, the Center for Vector-borne Infections (CVI), Uppsala University Hospital, Sweden–a collaboration between the Sections of Infectious Diseases and Clinical Microbiology–has been evaluating patients with persistent symptoms > 6 months after known or suspected tick-borne infection (TBI), where late-persistent LD or PTLDS had at some point been discussed as a conceivable cause, using standardized protocols and investigation procedures [14]. The impacts of other TBIs (e.g., anaplasmosis, babesiosis, rickettsiosis, neoehrlichiosis and bartonellosis) and manifestations, due to single or concomitant infections with LD, on the symptoms of these patients are also not fully elucidated but were part of the investigation [15–19].

Diagnosis of chronic LD has often been based on nonstandardized interpretation of serological tests, on nonvalidated or insufficiently validated laboratory tests, or on the clinical presentation alone [20].

Given the limitations of existing diagnostics for early LD or for patients with lingering symptoms such as PTLDS, and the absence of previous comparative data using protein biomarker profiling, the present exploratory study aimed to evaluate the feasibility of protein biomarker profiling as a diagnostic platform for TBI. We used two multiplex protein panels, that have previously been used in studies of fibromyalgia and chronic pain, thus allowing for simultaneous analysis of 92 inflammatory and neurology biomarkers [21, 22]. These biomarkers were analyzed in a cohort of patients with persistent symptoms after suspected exposure to TBI and then compared to 30 patients with laboratory-confirmed acute neuroborreliosis.

## Material and methods

### Patient cohorts and categorization

Two groups of patient cohorts (Cohort 1 and Cohort 2) were analyzed for biomarkers in serum and CSF, and the results were used for group-level comparison.

#### Original CVI patients

The patients in Cohort 1 were chosen from a pool of 224 patients, from a previously published study originating from the CVI clinic, where referred patients were enrolled and structurally investigated between October 2015 and December 2018 [14].

In these patients, having symptoms lasting longer than 6 months was a mandatory inclusion criterion. The other six inclusion criteria, of which patients should fulfill at least four, were > 18 years of age with suspicion of previous TBI based on: (a) previous tick exposure; (b) symptoms consistent with TBI; (c) laboratory findings (i.e., microbiological results), (d) previous treatments for TBI and/or (e) laboratory or clinical suspicion of co-infection with TBI other than LD.

Enrolled patients were examined by an infectious disease specialist and, besides a full medical history, underwent a panel of blood and CSF laboratory tests including hematological, biochemical, microbiological, and immunological analyses. The RAND-36 scale was used to allow patients to score their quality of life. Lumbar puncture was performed on patients with no contraindications. Antibodies in serum and CSF to *Borrelia* spp. had previously been tested, including Western Blot analysis as well as antibodies to *Anaplasma phagocytophilum*, *Bartonella henselae* and *B quintana*, *Rickettsia* spp. and TBEV, *Babesia divergens* and *B*. *microti* and PCR of *Rickettsia* spp. in CSF and *Candidatus* Neoerlichia mikurensis in serum. Most tests, including the immunological assays, had been performed using commercial kits as part of routine diagnostics at Uppsala University Hospital, Uppsala, Sweden.

As seen in Table 1 (S5 File), the 224 patients had previously been divided into 5 subgroups based on defined criteria and serological results; Group 0 represented 85 patients who met the CDC criteria for PTLDS [23]. Group 1 and 2 were seropositive for *Borrelia burgdorferi* s.l. (Bb s.l.) but lacked documented objective signs of LD. Group 1 (n = 31) included patients with antibodies only to Bb s.l.. Group 2 (n = 40) included patients with antibodies to Bb s.l. and any of the other TBIs. Group 3 and 4 were both seronegative for Bb s.l.. Group 3 (n = 32) represented patients with antibodies to any of the other TBIs, but not to Bb s.l.. Group 4 (n = 36) included patients with no antibodies to *Borrelia* s.l. or TBI (S5 File).

In brief, the results of that study showed that the PTLDS group did not differ significantly in any respect from the other subgroups, which either lacked previous objective evidence of borreliosis or even lacked signs of laboratory detectable exposure to LD, as well as that symptoms often categorized as "chronic Lyme disease" in the general debate could not uniquely be associated with LD or specifically with any of the other investigated TBIs. Most reported symptoms were fatigue related (70%), musculoskeletal (79%), neurological (82%) and neurocognitive (57%). Tick bites were recalled by 74% of patients. The RAND-36 score was significantly below that of the general Swedish population. However, approximately 20% of the total group of patients had elevated titers of myositis antibodies, a possible sign of autoimmunity, and 21% had slightly elevated fibrinogen values between 4.3–5.9 g/L, interpreted as a result of increased inflammatory activity. The outcome of that study is reported in a previous publication [14].

#### Cohort 1

From the original pool of 224 patients, the 158 patients who had undergone lumbar puncture and had both frozen serum and CSF samples available for protein biomarker analysis were selected for the current study. Of these 158 patients, hereafter referred to as Cohort 1, 55/85 represented patients from the PTLDS group in the previous study, 25/31 from Group 1, 32/40 from Group 2, 21/32 from Group 3 and 25/36 from Group 4. For statistical processing in the present study, the patients were regrouped based on previous clinical and laboratory findings. The new subgroups were: Group A—patients who met the PTLDS criteria (no. 55); Group B—seropositivity (IgG) for *Borrelia* that was confirmed by western blot (no. 78); Group C—seropositivity (IgG) for other TBIs (no. 64); and Group D—presence of inflammation (fibrinogen level > 4.3 g/l) (no.84). The patient’s expression of biomarker proteins in the respective subgroups A-D were compared group by group with the other patients in the cohort. The division and mutual distribution between groups is shown in S5 File.

The criteria used for PTLDS were in accordance with the Swiss and US case definitions of PTLDS, i.e., a clinically and/or laboratory documented episode of LD that, despite appropriate antibiotic treatment, leads within 6 months post-treatment to a constellation of disabling symptoms consisting of at least one of the following: fatigue, widespread musculoskeletal pain or cognitive problems [23]. Patients categorized as having PTLDS had been previously treated for LD based on objective signs (ECM, neuroborreliosis or acrodermatitis chronica atroficans. Seropositivity in combination with suspicion of LD, but without objective signs, was not considered sufficient to be assessed as PTLDS. However, seronegative patients presenting objective signs, such as ECM, were included if the other criteria were met.

#### Cohort 2

The second patient cohort, whose proteomic results, based on serum and CSF analyses from 30 patients with clinical and laboratory-confirmed neuroborreliosis and analyzed as part of routine diagnostics at Uppsala University Hospital Sweden, were compared with the 158 patients in Cohort 1. Between 1–3 weeks prior to diagnosis, those patients had been troubled by fever, fatigue, headache, sometimes also nausea, neck and back pain, or neuralgic pains. Cohort 1 and 2 were analyzed on different occasions. To normalize the cohorts to each other, eight samples from the sample matrix of Cohort 1 were included as bridge samples, i.e. re-run together with the samples from the group of patients with neuroborreliosis in Cohort 2. The samples in Cohort 2 had been previously tested for IgG and IgM antibodies in serum by ELISA (Euroimmun, Lubeck, Germany) against *Borrelia* spp. including *B*. *burgdorferi*, *B afzelii* and *B*. *garinii* and in CSF for anti-*B*. *burgdorferi* sensu latu with IDEIA Lyme Neuroborreliosis Kit (Oxoid Limited, former DAKO, Hampshire, UK).

#### Ethics statement

The present study, which was affiliated with the Department of Medical Sciences, Uppsala University, Sweden, was reviewed and approved by the Swedish Ethical Review Authority, Uppsala, Sweden (reg. no. 2015/249 and 2021/04739). The analyses were performed in accordance with relevant guidelines and regulations, and the participants gave their written informed consent.

### Protein biomarker panels

Two Olink Target Assay Panels, Olink Target 96 Inflammation and Olink Target 96 Neurology, based on proximity extension assay (PEA) techniques were performed in a 96-well plate to evaluate the expression of and measure the concentration of potential biomarkers on the Olink Multiplex platform (Olink Proteomics AB, Uppsala, Sweden), in each patient’s serum and CSF samples in the two cohorts. In each case, only 1 μL of the sample was used and treated with a pair of oligonucleotide-labelled antibodies that bind to their DNA target protein. When the two antibodies are in proximity, a new PCR reaction target sequence is formed, which is then detected and quantified using quantitative real-time PCR [21]. These assays each measure 92 potential protein biomarkers classified according to biological process, disease area, tissue expression and protein class based on widely used public access bioinformatic databases, including Uniprot, Human Protein Atlas, Gene Oncology (GO) and DisGeNET. All the analyzes and statistical processing of data were performed by the manufacturer Olink Proteomics AB, Uppsala Sweden (https://www.olink.com/products/targets/).

### Statistical analysis

The present study was designed to be exploratory, with given inclusion criteria and an unpredictable outcome, which precludes a power calculation. Instead, an arbitrary target of 158 + 30 patients was set based on the number of patients who could be included during the initial years of the study. After 3 years, that number had been achieved and the analysis of collected data began. The variation in the data between and within runs of the two panels was normalized to an arbitrary “Normalized Protein Expression (NPX) scale using the platform-specific “Olink NPX manager” software, which background corrects, log2 transforms and normalizes all samples. The statistical analyses were performed on normalized data and were expressed in NPX units on log2 scale, where a high value corresponds to high protein concentration and where a difference of one NPX corresponds to a doubling of protein concentration. The difference in mean NPX value for the biomarkers, between the groups compared in Cohort 1, was used for the calculations. T-tests were performed between the patients representing PTLDS (Group A) and the three other subgroups, i.e., with laboratory-confirmed signs of *Borrelia* spp. (Group B), other TBI (Group C) or inflammation (Group D). To claim statistical significance, a p-value was calculated using the Welch 2-sample t-test. An adjusted p-value was created using the Benjamini-Hochberg correction, thereby adjusting for multiple testing within each test, where an adjusted p-value less than 0.05 was considered statistically significant.

## Results

### Cohort 1 associated with PTLDS or persistent symptoms

Cohort 1 consisted of 158 patients, of whom 72 (46%) were men and 86 (54%) women. The overall median age was 54 years (range 20–79) and 53 years (range 18–80) for men and women, respectively. The number of patients and the subgroups A-D that were used for statistical processing are presented in S1 Table.

Of the 158 patients, 55 represented the PTLDS group. In total, 78/158 (49%) patients were seropositive (IgG) for *Borrelia* spp. confirmed by Western blot; 64/158 (41%) patients were IgG positive for any other TBI, and 84/158 (53%) had fibrinogen elevation as a marker for inflammation. Some patients were represented in more than one of the groups; 31/158 (20%) had antibodies for both *Borrelia* spp. and TBI; 43/158 (27%) were seropositive for *Borrelia* spp. and had concomitant signs of inflammation: 30/158 (19%) were seropositive for TBI and had signs of inflammation: 18/158 (11%) were seropositive for both *Borrelia* spp. and TBI and showed signs of inflammation. Six of the patients in the PTLDS group had elevated levels of antibodies in CSF, but no signs of current infection and 16 patients in total had detectable myosit antibodies. The distribution between variables and subgroups is illustrated in S1 Table.

### Protein biomarker distribution in Cohort 1

Proteins with at least 25% detectability were included in the further analysis. As a result, 26/184 (14%) biomarker assays were excluded from the CSF assays and 6/184 (3%) assays from the serum assays because the signals were too low. The results for all remaining samples, 158/184 from CSF and 178/184 from serum, were assessed using a boxplot and unsupervised principal component (PCA) analysis of the NPX distribution, and the samples that differed from others in the box plots regarding low spread of the signal–or low or high signal, or that deviated in the PCA analysis and were assessed as outliers, were excluded from the compilation. These consisted of nine CSF samples in the Olink 96 Target Inflammation and Neurology assays, resulting in 149/184 remaining CSF samples. The corresponding figures for the serum samples were four and three samples, respectively, which differed in the box plot but did not deviate in the PCA analysis, which is why they were retained (178/184) in the continued analysis.

### Protein biomarker signature in Cohort 1

**Fig 1:**
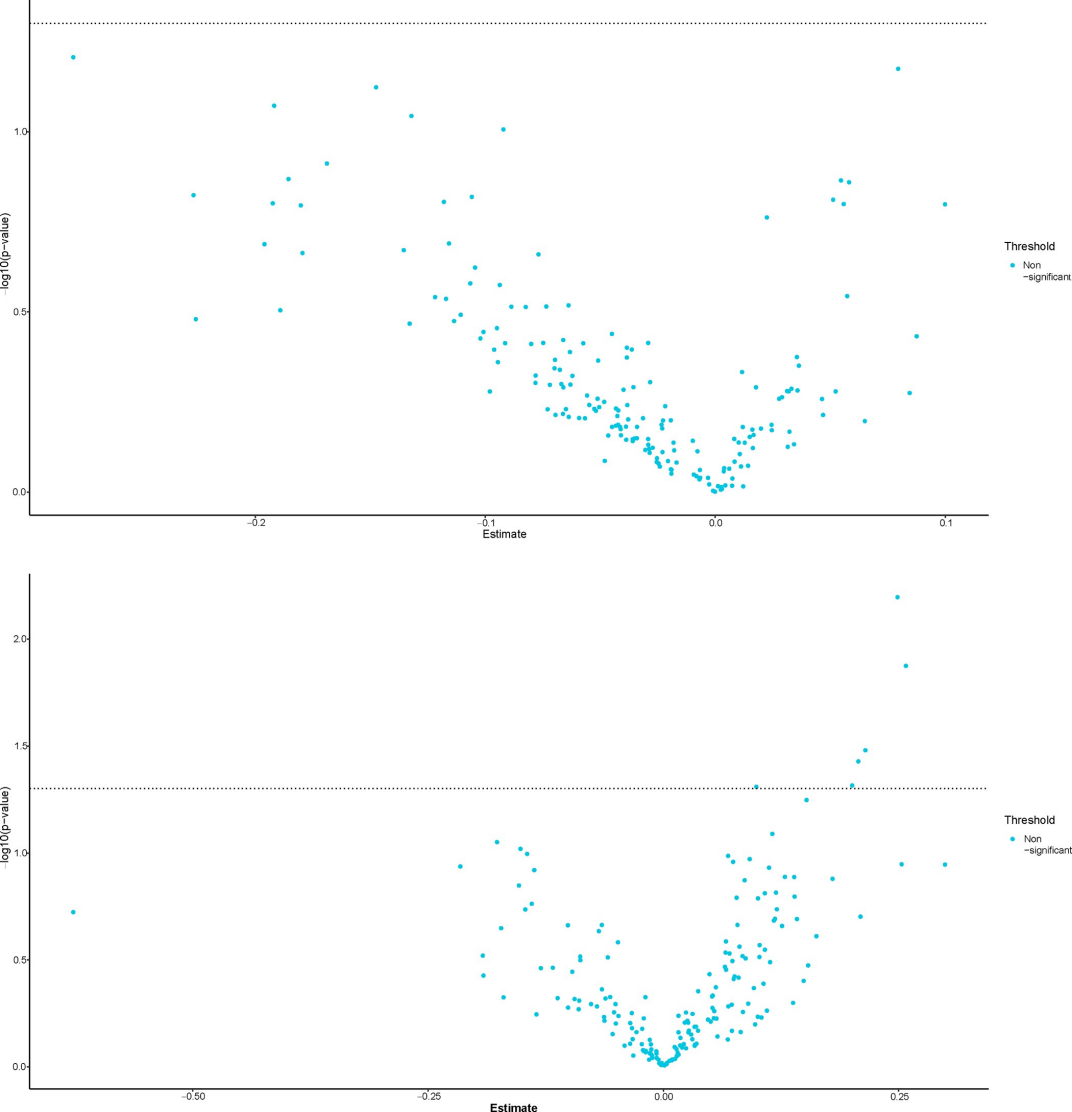
a-b. Volcano plots of differentially expressed proteins between PTLDS and the other CVI patients for the CSF (a) and serum (b) samples. X-axis shows the mean difference between the two groups, and y-axis shows the -log10(p-value), where the p-value is given by a Welch 2-sample t-test. All p-values were adjusted for multiple testing according to the Benjamini and Hochberg procedure. For both sample types, no proteins were found to be nominally significant.

**Fig 2:**
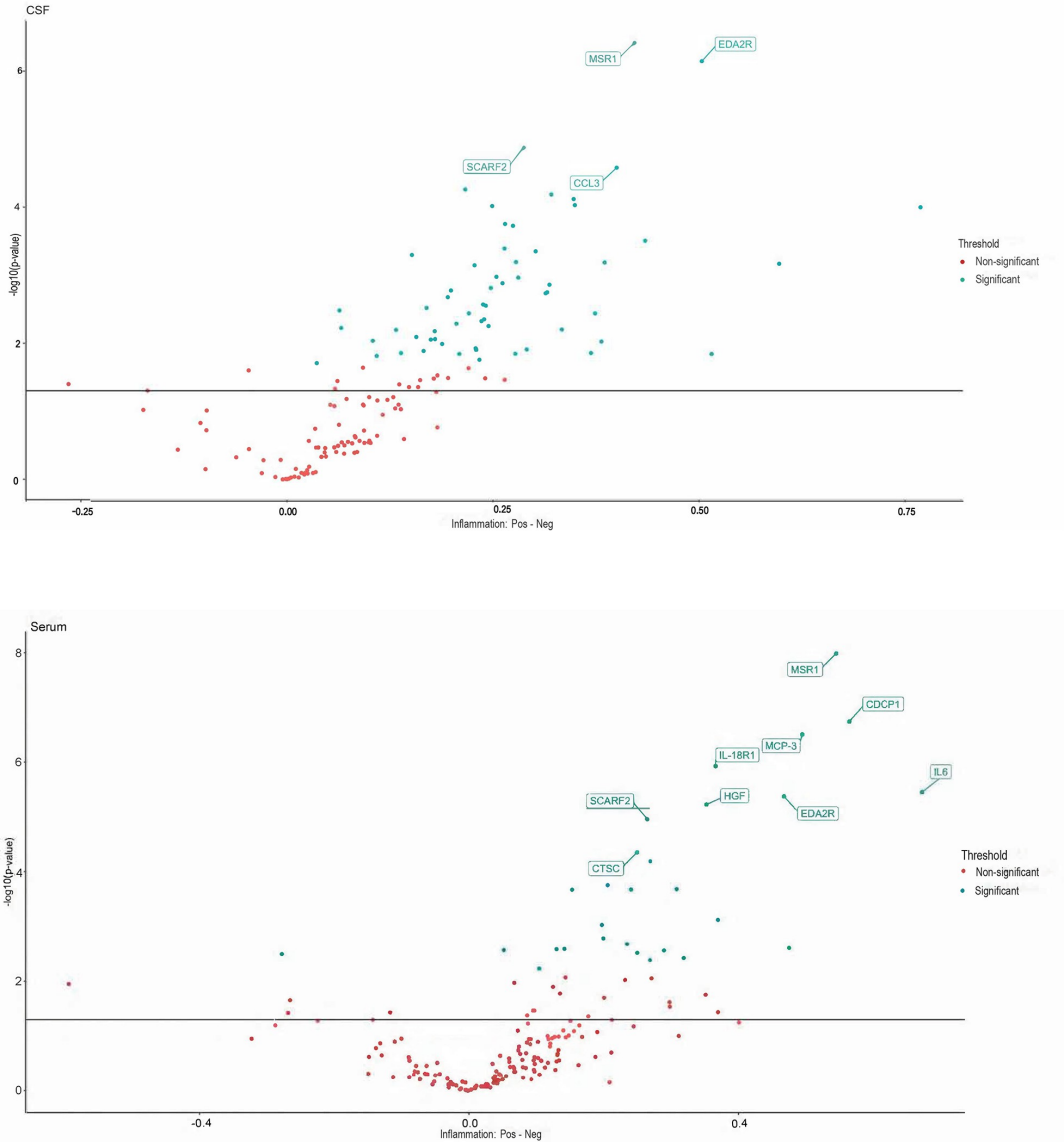
a-b. T-test between patients with and without inflammation in CSF (a) and serum (b). 61/149 proteins were significantly different between patients with and without inflammation in CSF and in serum 29/178 proteins differed significantly. Proteins on the positive x-axis have higher NPX values in the Pos group. Proteins on the negative x-axis have higher NPX values in the Neg group. Proteins with p-value less than 0.001 are labeled.

**Fig 3:**
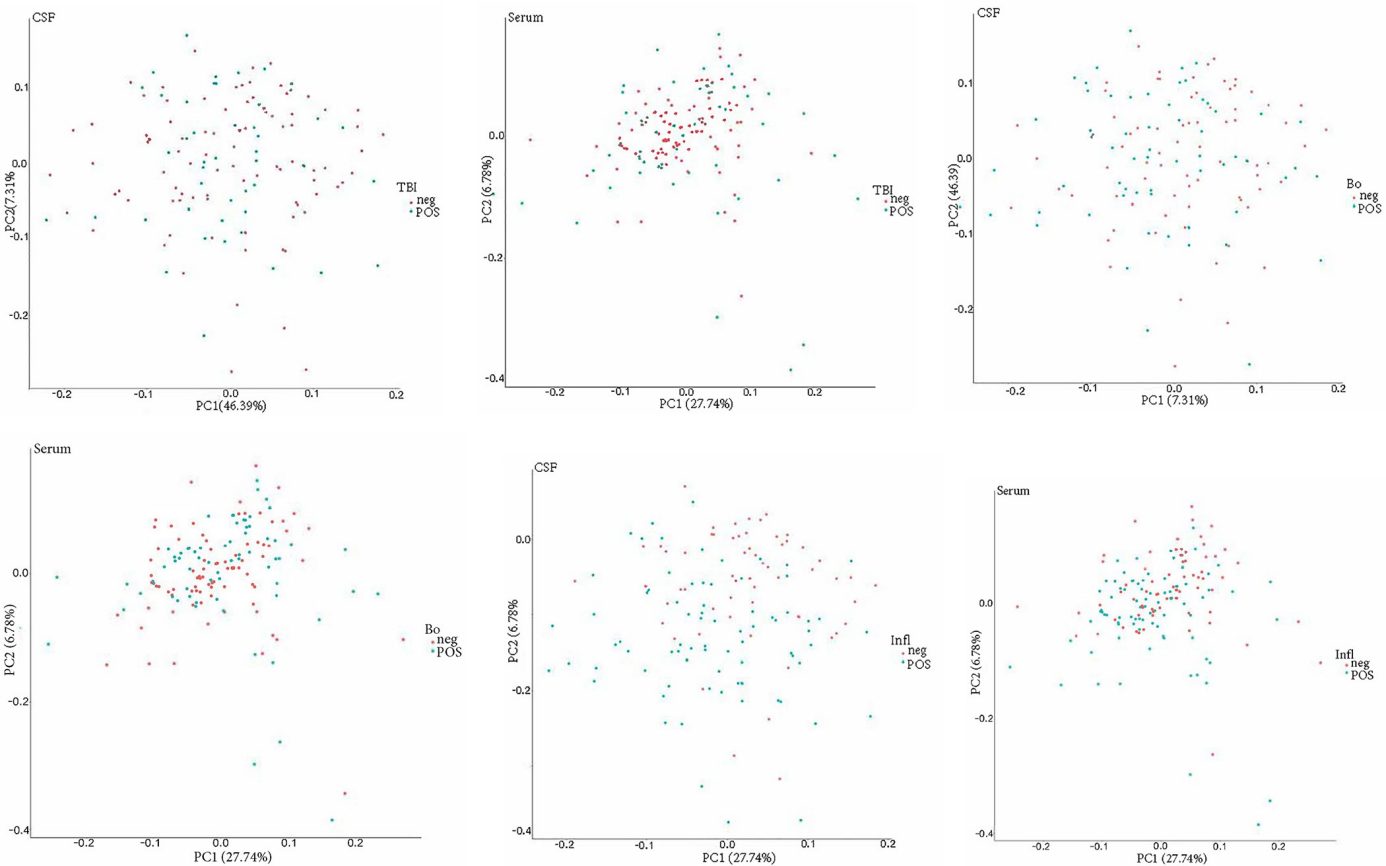
a-f. Unsupervised principal component analysis for CSF (a, d, e) and serum (b, d, f). PCA plots for the different groups of patients who in addition to *Borrelia* were serologically positive or negative for tick-borne disease (TBI), furthermore the group serologically positive / negative for *Borrelia* spp. (Bo) and patients with elevated or normal fibrinogen as marker för inflammation (Inf) was generated, but no clear separation was seen in any of the PCA plots.

T-test of the PTLDS patients (Group A) for both sample types (CSF, serum), showed no nominally significant proteins when comparing the PTLDS group to the other patients in Cohort 1 (Fig 1A and 1B). In t-test determination of the three other subgroups (B-D), 0 of 149 proteins were significantly different in CSF, except in patients for whom previous biochemical analysis had shown an elevated fibrinogen value as a biomarker of inflammation. Among these patients, 61 of 149 proteins were significantly different in CSF and 29/178 in serum, compared to patients without inflammation. Proteins with p-values <0.001 consisted of SCARF2, CCL3, MSR1 and EDA2R. T-tests between patients with and without inflammation in serum showed 29/158 proteins that were significantly different, of which nine proteins (CTSC, SCARF2, HGF, EDA2R, IL-8, IL-18R1, MCP-3, CDCP-1 and MSR1) demonstrated p-values <0.001 (Fig 2A and 2B). Sixteen proteins were found to be significant (p< 0.05) in both CSF and serum (MSR1, IL-18R1, IL6, EDA2R, HGF, SCARF2, CTSC, SCARA5, SIGLEC1, NRP2, EFNA4, SCARB2, EZR, LAYN, CLM-1 and CTSS). No significant differences in the expression of biomarker proteins were found depending on whether the patient was seropositive or negative for the *Borrelia* spp. or other TBIs. When using PCA for CSF and serum, no clear separation or subgroup of samples was seen (Fig 3A–3F). In a further analysis with unsupervised hierarchical complete linkage clustering of CSF and serum, aimed at identifying similar clusters and subgroups of samples, three clusters were used in CSF and serum (Cluster-*Borrelia* 1–3, Cluster-TBI 1–3 and Cluster-Inflammation 1–3), but no obvious correspondence to *Borrelia* spp. other TBIs or inflammation was seen, though some potentially interesting subgroups, which could be investigated further, were revealed. The data used for statistical tests and the results can be found in the supporting information S1 and S2 Files.

### Protein biomarker signature associated with acute neuroborreliosis in Cohort 2

**Fig 4:**
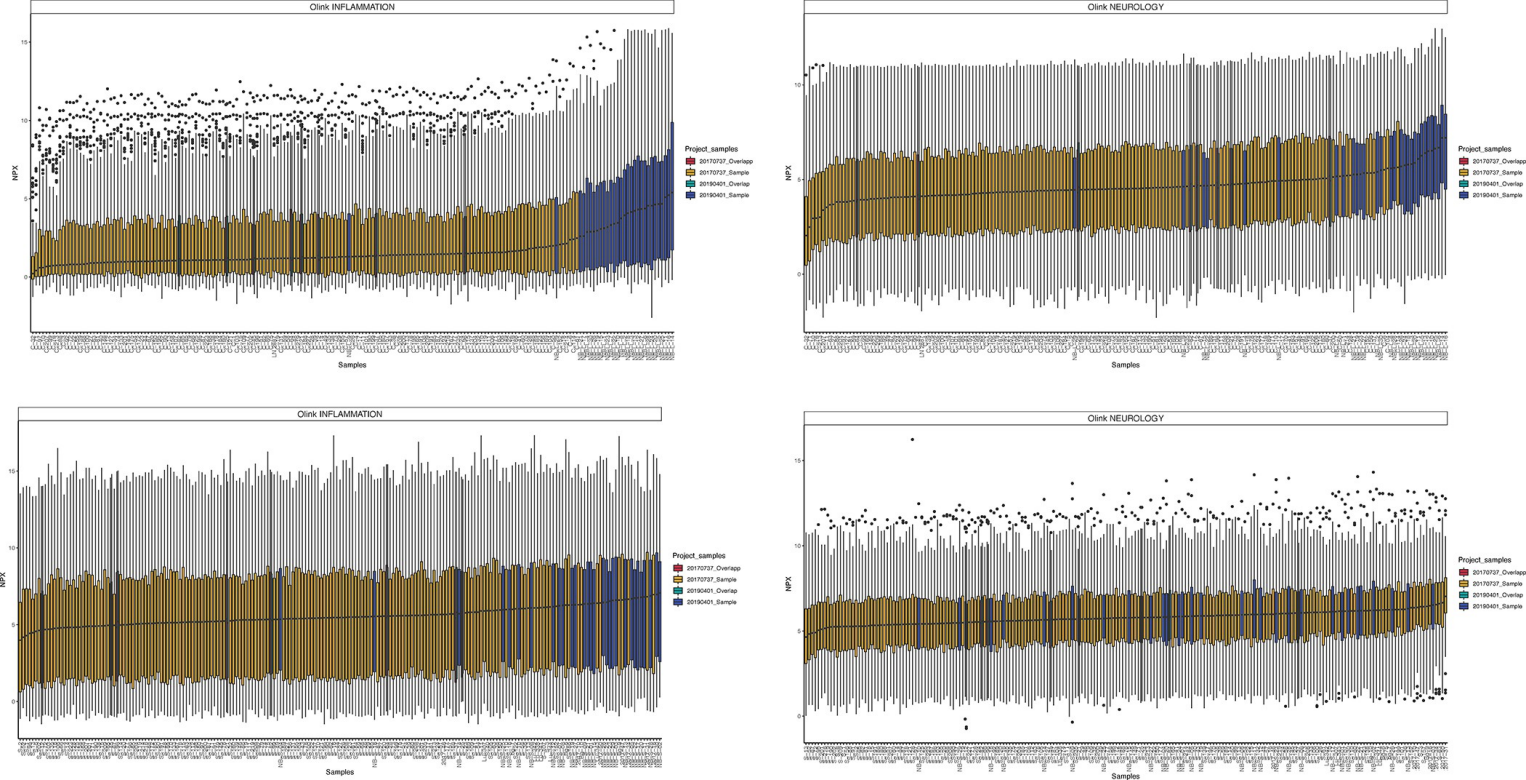
a-d. Boxplots after normalization. The boxplots show the distribution in CSF (a, b) and serum (c, d) on Olink 96 Target Inflammation and Neurology panels after normalization of NPX values for CVI patients and patients with neuroborreliosis.

Cohort 2 consisted of 30 patients (14 men and 16 women) who were sampled between Oct 2016 and April 2019. The median age for men was 40 years (range 3–84 years) and for women 66 years (range 5–78 years). Four of the patients were children, one boy and three girls, between three and eight years of age. Proteins with less than 25% detectability after normalization and combination of datasets were excluded from further analysis. This resulted in 150 proteins for CSF and 179 proteins for serum analysis. After normalization, a subset of samples from Cohort 1 were selected that were serologically positive for *Borrelia* spp. and negative for other TBIs; these bridge samples were compared with the group of patients with “ongoing *Borrelia*” in Cohort 2. It was obvious that the NPX values were much higher in patients with ongoing *Borrelia* compared to both the bridge samples analyzed on the same sample plate and to all sample results from previous analysis of the entire Cohort 1. The NPX levels for the bridge samples were rather similar between the two cohorts, leading to just a small adjustment between the analysis occasions, mainly for the CSF samples. Boxplots of the distribution after normalization are shown in Fig 4A–4D.

### Significantly expressed proteins in Cohort 2

**Fig 5:**
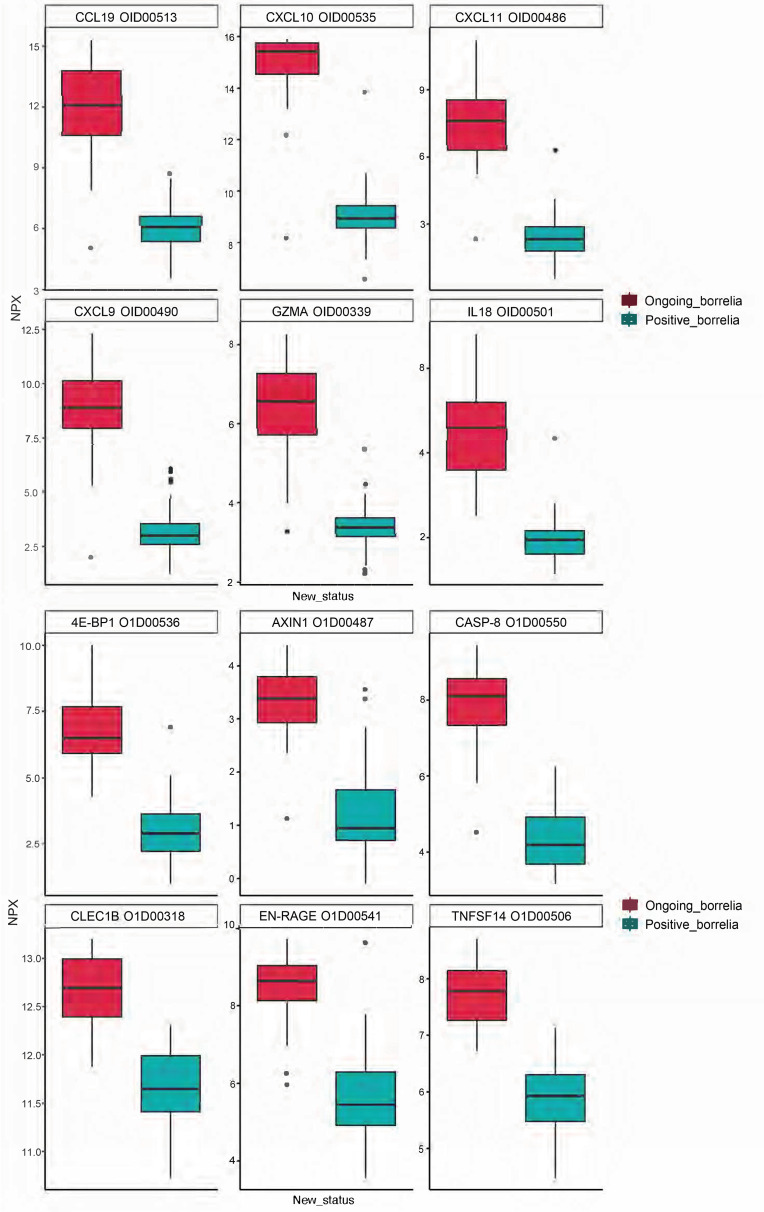
a-b. Box diagrams with the top 6 most significant proteins in CSF (a) and serum (b). The proteins represent those most different between CVI patients and patients with ongoing neuroborreliosis.

**Fig 6:**
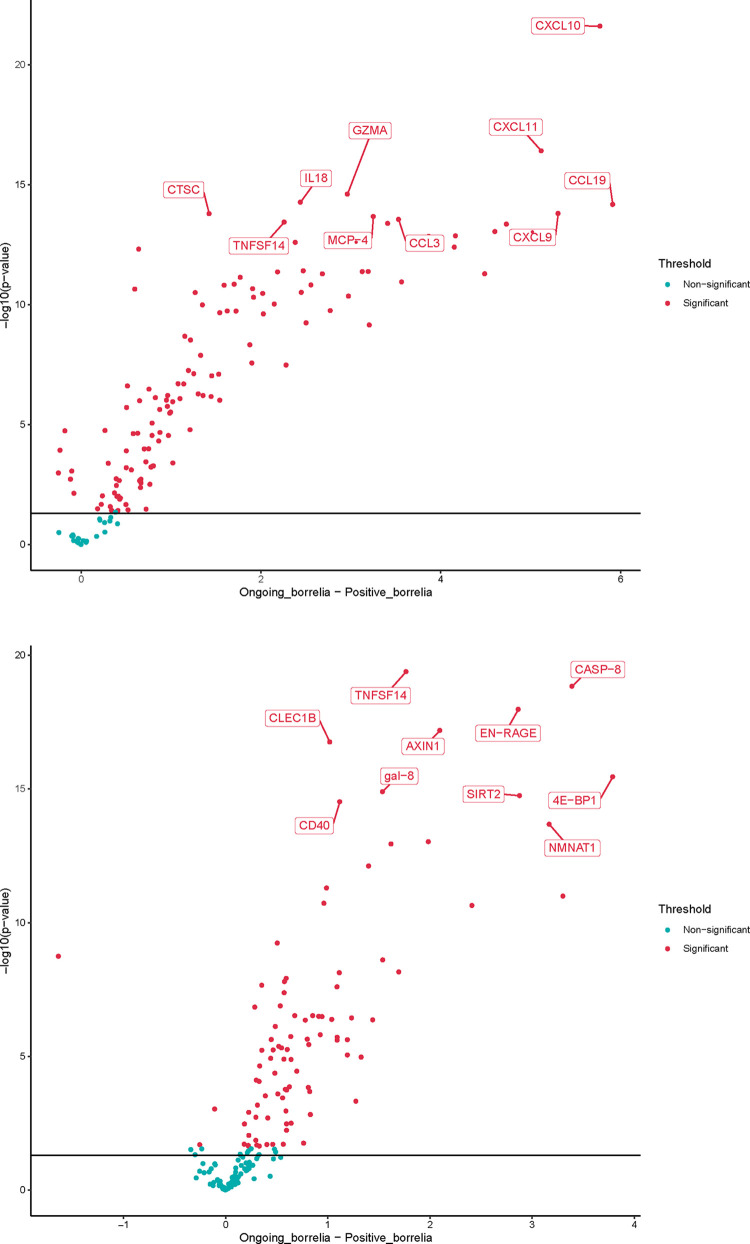
a-b. Volcano plots of the CSF (a) and serum (b) samples for CVI patients and those with on-going neuroborreliosis. The plots show the relation between estimate and p-value. On the x-axis is the NPX difference between the two groups. Y-axis shows the -log10(p-value). The solid line is the raw p-value 0.05 cutoff and the red proteins have a significant adjusted p-value below 0.05.

The number of proteins that were significantly different between the cohorts and that were mainly expressed in patients with neuroborreliosis were, in CSF, 125 of 148 proteins and, in serum, 93 of 174 proteins. Of the most significant proteins that differed between the cohorts and were expressed in Cohort 2 in CSF at the p<0.001 level, we can note CCL19, CXCL10, CXCL11, CXCL9, GZMA and IL18 and in serum 4E−BP1, AXIN1, CASP−8, CLEC1B, EN−RAGE and TNFSF14. The differences are illustrated in Fig 5A and 5B and by Volcano plots showing the relation between estimate and p-value (Fig 6A and 6B). The t-test and normalized data for CSF and serum can be found in the supporting information S3 File.

## Discussion

This is the first extensive inflammatory and neurology biomarker profile study that has been conducted on patients with persisting symptoms after presumed tick-bite exposure. The biomarkers that were detected in Cohort 1 had no association with whether *Borrelia* exposure could or could not be detected. Moreover, in patients suffering from acute neuroborreliosis, in Cohort 2, several significantly expressed proteins were detected in both CSF and serum compared with Cohort 1.

LD is very common throughout Europe as well as in the United States [24]. Appropriate antibiotic treatments of these patients, have been well described, as well as cures in most cases [12]. However, between 0.5 to 13.1% of LD patients have reported nonspecific symptoms 6 month or later following treatment. Whether these unexplained symptoms are related to prior LD remains unclear. According to the definition presented by the Infectious Disease Society of America (IDSA) in 2006, PTLDS is a debilitating condition characterized by chronic fatigue, pain, and cognitive difficulties that last 6 months or more despite treatment [16, 25, 26]. The underlying mechanism responsible for PTLDS symptoms, as well as a reliable diagnostic tool, has remained elusive [12, 27–29]. In the present study, we examined the association between specific findings of protein markers in different categories and subgroups of patients. When comparing patients who met or did not meet the criteria for PTLDS, no significant differences in biomarker profiles were seen. Only in patients with persisting symptoms and with laboratory findings of inflammation in the form of moderately elevated fibrinogen levels, were several proteins in CSF and serum found to be significantly differentially expressed. Of these, 16 were found to be significant (p< 0.05) in both CSF and serum, regardless of the serological status of *Borrelia* or TBI. These proteins were MSR1, IL-18R1, IL6, EDA2R, HGF, SCARF2, CTSC, SCARA5, SIGLEC1, NRP2, EFNA4, SCARB2, EZR, LAYN, CLM-1 and CTSS, which are represented in several different disease areas including pulmonary, cardiovascular, neurological, skeletal, hemic, lymphatic, metabolic, and inflammatory diseases such as rheumatic disease, systemic sclerosis, and Sjögren’s syndrome, but also demyelination, neuropathies, and prostate cancer (S2 and S3 Tables). The tissue expression of the proteins is represented by several different organs and cell types such as macrophages, leukocytes, bone marrow, liver, lung, heart, brain, skeletal muscle, pancreas, intestinal mucosa and synovia (S2 Table). Detection of these proteins does not explain why the persistent symptoms, including patients with PTLDS, occur, but may provide early insights into the disease processes that are modulated as a function of disease and facilitate the identification of protein candidates for further study. However, it is unclear whether increased protein levels represent a natural response to disease, rather than a causative factor leading to disease. The causal direction between disease and disease-related protein biomarkers could possibly be better resolved by also performing a two-way analysis using the Mendelian Randomization method [13].

It is currently difficult to associate the persistent symptoms in this group of patients with any specific syndrome or process, and if we are to do so further studies are needed. When comparing the results for the CVI patients in Cohort 1 with the results for a group of patients with similar symptomatology, such as fibromyalgia and chronic pain, another inflammatory profile is seen in both cases compared with the CVI cohort [22]. The top five proteins that distinguished fibromyalgia patients from plasma controls were in serum STAMBP, SIRT2, CD40, AXIN1 and IL-7 and in CSF (CCL19, CCL23, IL-18, CX3CL1, FGF19, CXCL10, CCL11). For patients with chronic widespread pain four proteins were important for group discrimination both in CSF and in plasma (CXCL6, LAPTGF-beta-1, CXCL5, MCP-2). The differences between the groups may reflect different disease processes. A proteome analysis has previously also been conducted on CSF of subjects with PTLDS vs those with chronic fatigue syndrome [30]. The samples were analyzed using high-resolution mass spectrometry (MS), coupled with immunoaffinity depletion methods to reduce protein-masking by abundant proteins. The example of proteins elevated in abundance in the two disease conditions, compared to normal, but at different levels, applies above all to the complement cascade-related proteins such as C1S, C4B, C1QB, C1QC. There were also several proteins solely identified in one condition and representing proteins involved in cellular metabolism, energy homeostasis, secretory and signaling functions. Gene biomarkers that represent, for example, inflammatory processes, cell-cell signaling, cellular interaction, uptake and development are also presented in the current study, but the findings need further validation to make an accurate comparison with the proteins reported in the respective studies. The most significant proteins (p <0.001) that differed between the Cohort 1 and 2 were CCL19, CXCL10, CXCL11, CXCL9, GZMA and IL18 in CSF, and 4E - BP1, AXIN1, CASP-8, CLEC1B, EN—RAGE and TNFSF14 in serum. None of these proteins was significantly expressed in Cohort 1, they mainly represent disease areas such as infectious, inflammatory, pulmonary, hepatic, metabolic, hemic, lymphatic and cardiovascular diseases and are mainly expressed in cells listed in Table S3 File. Other studies of patients with LD have shown similar findings as seen in Cohort 2, with upregulation of T-cell specific mediators, where the T-cell chemoattractants CXCL9, CXCL10 and CCL19 were significantly elevated in serum during an ongoing infection, but largely returned to normal levels following treatment [31]. It has also been reported that there is a selective and coordinated increase in T-cell chemoattractants in acute LD, and in contrast to the chemoattractants mentioned above, the levels of CCL2-5, CCL7-8, CCL11 or CXCL12 are not affected compared to matched controls [32]. An additional study examined the serum proteome for specific acute phase proteins in early LD [33]. Ten proteins, with significantly altered serum levels in patients at the time of diagnosis were identified that distinguished LD patients from healthy controls. These proteins primarily represented innate immune response proteins or proteins specific to liver, skin or white blood cells, i.e biological processes that also are represented in the findings related to Cohort 2. Interestingly, in mouse models, the liver is a well-defined site for *B*. *burgdorferi* dissemination and high levels of CXCL9 and CXCL10, which were also detected in Cohort 2 and have been shown to be closely related to the extent of liver involvement as measured by blood liver enzyme levels [34]. The other proteins found in Cohort 2, in patients presenting neuroborreliosis, represent biomarkers that are often activated in inflammation or immune response in infection, activation of T and NK cells and cell death and that probably do not represent a protein profile specific to acute LD (S6 File). The clinical findings and inflammatory response may also differ between prepubertal children and adults [35]. In the present study, the majority of patients with neuroborreliosis were adults, except for four children (13%) 3–8 years of age. No subgroup could be distinguished in the results attributable to these children, so the results for the entire group of neuroborreliosis patients were probably not affected in any direction. In the present study, CXCL13 –which was part of the neurology panel and has been proposed as a biomarker of neuroborreliosis, especially in children, and is released by monocytes following exposure to the outer surface proteins from *Borrelia–*was in the present study not identified among the investigated patients [36].

## Conclusions

In summary, although this is the first comprehensive inflammatory and neurological biomarker profile study, no differences in biomarker profiles were detected between patients with PTLDS and patients with similar persistent symptoms but who did not meet the PTLDS criteria, regardless of laboratory-confirmed previous exposure to *Borrelia* or other TBIs. However, there was a difference in the biomarker profile expressed in these patients compared to patients with confirmed acute neuroborreliosis, which does not support the view that PTLDS reflects an ongoing *Borrelia* infection. Further studies are needed to understand and assess the usefulness of biosignatures from patients with PTLDS before they can be applied in a clinical setting. An indication of potentially interesting subgroups in both CSF and serum was also revealed, although not further defined at this stage, but may be of interest for further investigation.

## Supporting information

S1 Table Previous grouping of the original CVI patients (0–4) and after re-grouping of patients in Cohort 1 (A-D). The groups represents both the groups of patients from the previous CVI study (Groups 0–4) and the regrouping of Cohort 1 (Group A-D) in the current study for the statistical calculations based on the outcome of key parameters, i.e., patients who met the criteria for PTLDS, serological outcomes and laboratory signs of inflammation.
(XLSX)

S2 Table Background information for the biomarkers expressed in Cohort 1. The table summarize the correlation of the expressed biomarkers to biological process, tissue expression, disease area and associated diseases among CVI patients in Cohort 1.
(XLSX)

S3 Table Background information for the biomarkers expressed in Cohort 2. The table summarize the correlation of the expressed biomarkers to biological process, tissue expression, disease area and associated diseases among patients with ongoing neuroborreliosis in Cohort 2.
(XLSX)

S1 File Cohort 1. Statistical test data and results of t-test, and cluster calculations of CSF and serum.
(XLSX)

S2 File PTLDS group. Statistical test data and results of t-test of CSF and serum.
(XLSX)

S3 File Cohort 1 and 2. T-test and normalized CSF and serum data.
(XLSX)

S4 File (XLSX)

S5 File (XLSX)

S6 File (XLSX)

## Abbreviations

CSF: cerebrospinal fluid
LD: Lyme disease
NPX: normalized protein expression
PTLDS: post-treatment Lyme disease syndrome
TBI: tick-borne infection
TBEV: tick-borne encephalitis virus
